# Molecular Insights Into the Sensory Adaption of the Cave‐Dwelling Leech *Sinospelaeobdella wulingensis* to the Karst Cave Environment

**DOI:** 10.1002/ece3.70877

**Published:** 2025-01-18

**Authors:** Xi Wen, Haiyang Xiang, Mengqing Zhang, Aoran Yan, Dongqing Xiang, Jie Zou, Yue Zhang, Xinglong Huang, Zhixiao Liu

**Affiliations:** ^1^ Hunan Provincial Key Laboratory of Ecological Conservation and Sustainable Utilization of Wulingshan Resources College of Biology and Environmental Sciences, Jishou University Jishou Hunan China

**Keywords:** cave‐dwelling leech, ionotropic glutamate receptor, mechanosensitive ion channel, *Sinospelaeobdella wulingensis*, transcriptome

## Abstract

Karst caves are a unique environment significantly different from the external environment; adaptation of cave‐dwelling animals to the cave environment is often accompanied by shifts in the sensory systems. Aquatic and terrestrial leeches have been found in the karst caves. In this study, we conducted a transcriptome analysis on the cave‐dwelling leech *Sinospelaeobdella wulingensis*. A total of 29,286 unigenes were obtained by assembling the clean reads, and only 395 genes are differentially expressed in winter and summer samples. Two piezo‐type mechanosensitive ion channels (Piezos), eight transient receptor potential channels (TRPs), and six ionotropic glutamate receptors (iGluRs) were identified in the transcriptome. These channels/receptors are transmembrane proteins sharing conserved structural features in the respective protein families. SwPiezo1 shares high identity with Piezos in non‐caving leeches. SwiGluRs are conserved in protein sequence and share high identities with homologous proteins in other leeches. In contrast, SwTRPs belong to different subfamilies and share diverse identities with TRPs in other species. Gene expression analysis showed that two SwPiezos, five SwTRPs, and one SwiGluR are abundantly expressed in both winter and summer samples. These results suggest that SwPiezos, SwTRPs, and SwiGluRs are candidate sensory channels/receptors that may have roles in mechanosensory and chemosensory systems. High expression levels of Piezo and TRP genes imply a mechanosensory adaptation of *S. wulingensis* to the hanging living style in caves. Furthermore, enrichment of sensory genes in the oral sucker indicates the important role of this tissue in response to environmental stimuli. Similar gene expression profiles in winter and summer samples imply a stable physiological status of *S. wulingensis* in the cave environment.

## Graphical abstract

We conducted a transcriptome analysis on the cave‐dwelling land leech *Sinospelaeobdella wulingensis*. Piezos, TRPs, and iGluRs may have roles in mechanosensory and chemosensory systems of this leech. Similar gene expression profiles in winter and summer samples imply a stable physiological status of *S. wulingensis* in the isolated cave environment.

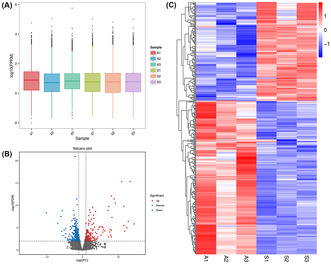

## 1 Introduction

Karst caves are a unique environment characterized by malnutrition, low air mobility, long‐term darkness, and a stable microclimate (Zhao et al. 2024). Cave‐dwelling animals surviving in the karst caves highly rely on their non‐vision sensory systems, extreme starvation hardiness, excellent nutrient utilization efficiency, and many other cellular, physiological, and morphological characteristics (Luo et al. 2018; Lipovšek et al. 2019). In karst caves, most of the organic materials basically come from the surface environments. These survival materials are generally carried by running water and troglophiles, especially bats, providing the primary nutrient resource for the trophic levels in the caves (Tanalgo, Oliveira, and Hughes 2022; Garduño‐Sánchez et al. 2023). In the subtropical area, a great amount of bats habitat in the karst caves and bring a huge amount of organic substances to the caves during the breeding season. However, the resident cave bats will hibernate in the caves during winter, while the migratory bats go away to their winter destinations (Haest et al. 2021). Therefore, many cave ecosystems demonstrate seasonal dynamic changes related to the surface world. Animals living in these caves highly rely on the limited organic substances (D'Angeli et al. 2017). Starvation hardiness and efficient chemosensory systems as possible adaptations to opportunistic diets were observed in many cave arthropods (Whitten 2009; Luo et al. 2018; Balart‐García et al. 2021).

The karst caves consist of multi‐scale subterranean spaces, such as tunnels and halls, with a complex three‐dimensional structure (Ma et al. 2023). For the troglobites, adaptation to these unique environments is often accompanied by shifts in their sensory systems. Evidence in the literature indicated that the chemosensory systems in the cavefishes are enhanced in the blind species in comparison with the surface species (Roberts et al. 2018). Cave‐dwelling insects rely heavily on chemical and tactile information for locating mates and food resources (Fea, Mark, and Holwell 2018). Transformations of sensory structures on the antennae of cave insects were implied to be compensated for the degenerate vision ability (Merivee et al. 2003; Fea, Mark, and Holwell 2018; Luo et al. 2019). Sensory proteins involved in mechanosensory systems, such as Piezo‐type mechanosensitive ion channels (Piezos) and transient receptor potential channels (TRPs), and chemosensory systems, such as odorant receptors (ORs) and ionotropic glutamate receptors (iGluRs), are abundantly expressed in the sensory organs (Xiao et al. 2017; Dhakal and Lee 2019; Wang et al. 2020). In the troglobitic beetle *Speonomus longicornis*, duplication and loss of chemosensory genes indicated the evolution of the sensory system at molecular and genetic levels in response to the unique cave environment (Balart‐García et al. 2021). The isopod crustacean *Asellus aquaticus* has both surface and cave forms, and the cave individuals lose eyes and body pigment but have longer antennae when compared with the surface individuals (Mojaddidi et al. 2018).

Animal sensory channels/receptors define a large superfamily of transmembrane proteins that function as sensors of internal and external stimuli and play important roles in a wide range of biological processes and behaviors (Fang et al. 2021; Zhou and Martinac 2023). The Piezo channels are nonselective cation channels that are sensitive to mechanical stimuli. In higher vertebrates, Piezo1 is expressed ubiquitously in most tissues, whereas Piezo2 is expressed more specifically in the peripheral sensory neurons (Shin et al. 2023; Aragona et al. 2024). Piezo1/2 form a functional homotrimer structure comprising a central ion‐conducting pore and three peripheral blades, which confer exquisite mechanosensitivity to Piezo channels (Jiang et al. 2021). In invertebrates, Piezos also play a role in mechanotransduction involved in locomotion regulation, internal organ sensation, cell differentiation, cellular immune, and many other biological functions associated with neural and cellular sensation of stretch (Gottlieb and Sachs 2012; He et al. 2018; Hu et al. 2019; Wang et al. 2020). TRPs comprise a large group of related cation channels that display great diversity in the specific modes of activation and cation selectivities (Montell 2005). These proteins typically share six transmembrane segments and are divided into a range of subtypes, each likely possessing distinct biophysical attributes (Dhakal and Lee 2019; Himmel and Cox 2020). TRPs generally function as homologous or heterologous tetramers, and several members of this group have well‐known functions in chemosensation, thermosensation, visual transduction, and mechanosensation (Montell 2012; Diver et al. 2022). The iGluRs, which are a large family of membrane receptors, have been identified as neurotransmitter receptors in synaptic transmission and critical sensory receptors for environmental stimuli (Lee et al. 2017). Animal iGluRs are divided into different functional classes, namely α‐amino‐3‐hydroxy‐5‐methyl‐4‐isoxazolepropionic acid (AMPA) receptors, kainate receptors, *N*‐methyl‐d‐aspartate (NMDA) receptors, and GluD receptors (Krieger, Bahar, and Greger 2015; Knecht et al. 2016). Subunits within each functional class can assemble as functional homomers and heteromers guided by specific sets of rules and function as ligand‐gated receptors that mediate a large portion of neurotransmission, chemosensation, thermosensation, and hygrosensation (Brockie and Maricq 2003; Hansen et al. 2021). These sensory channels/receptors play important roles in sensory systems and are essential for animal survival, feeding, reproduction, and many other biological processes.

**FIGURE 1:**
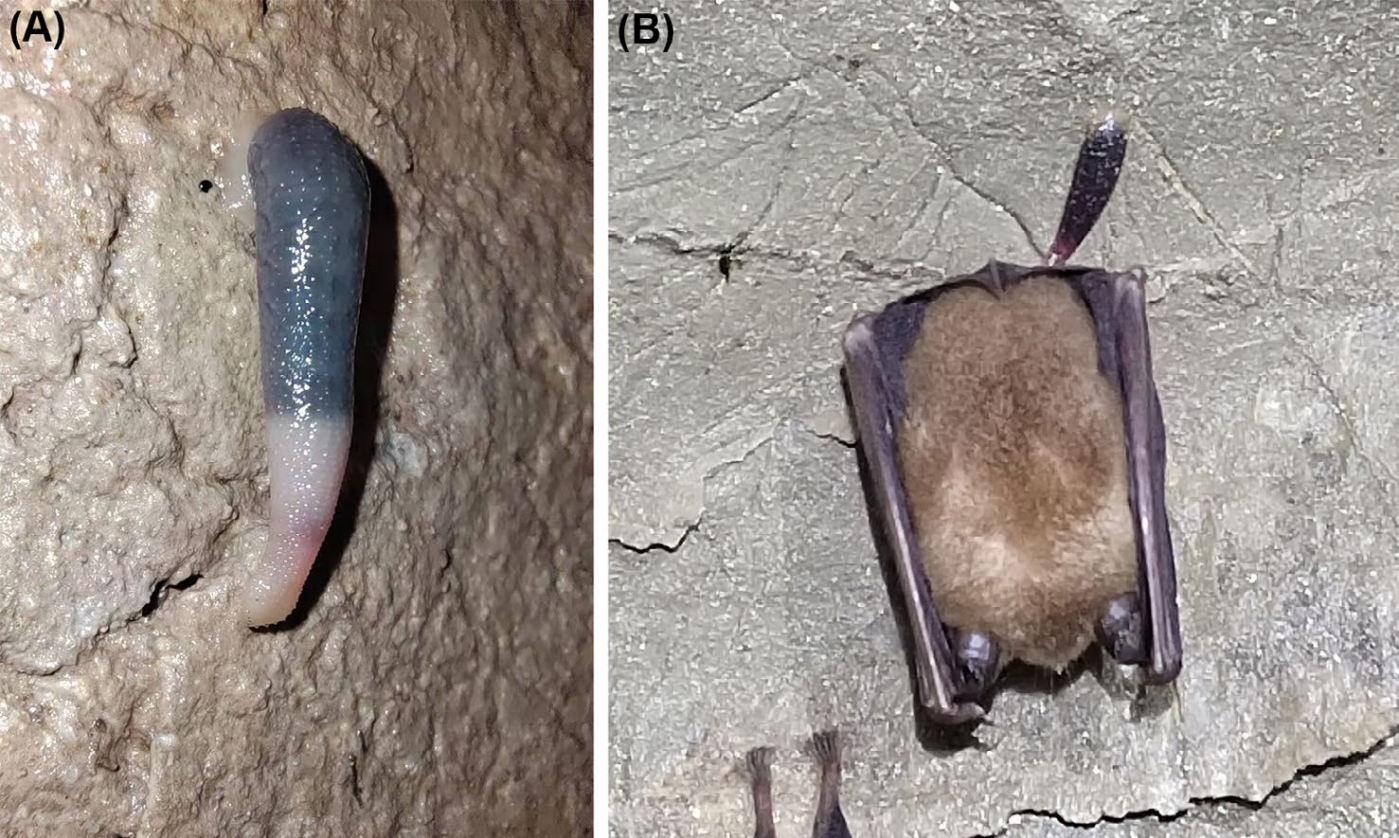
*Sinospelaeobdella wulingensis* in a karst cave. (A) *S. wulingensis* individual in Tangle Cave, Hunan Province, China. (B) *S. wulingensis* bloodsucking on the wing of a bat.

Aquatic and terrestrial leeches have been collected in karst caves. *Croatobranchus mestrovi*, *Erpobdella borisi*, and *Trocheta ariescornuta* were found in subterranean water environments (Kerovec, Kučinić, and Jalžić 1997; Sket et al. 2001; Cichocka et al. 2015; Grosser, Barjadze, and Maghradze 2021). The leech *Batracobdella algira* was found in different water and terrestrial habitats, including the subterranean ones (Lunghi et al. 2018). In the subterranean habitats, *B. algira* was found parasitizing frogs and salamanders, suggesting the transition of cave leeches to the troglophilous hosts (Manenti et al. 2016). Species of the land leech genus *Sinospelaeobdella* inhabit the karst caves and have been described as opportunistic blood‐feeders on cave bats (Yang, Mo, and Wang 2009; Huang, Liu, et al. 2019; Huang, Titus, et al. 2019). Our previous study collected *Sinospelaeobdella wulingensis* (Hirudinida: Haemadipsidae) in karst caves and described this species with a unique living style, hanging on the cave roof and walls (Figure 1) (Huang, Liu, et al. 2019). *Sinospelaeobdella wulingensis* has an anterior (oral) sucker and a posterior (caudal) sucker and moves on the cave roof in a geometer style. The mouth and eyes are located on the center and edge of the oral sucker, respectively, indicating the important roles of the oral sucker in sensory and feeding. *Sinospelaeobdella wulingensis* has demonstrated morphological adaptation to the cave environment, including fading of surface pigment and extensive oral and caudal suckers (Huang, Liu, et al. 2019). However, the molecular basis for the mechanosensory and chemosensory functions in this troglobite, which adapts to the unique hanging living style in the karst caves, still remains unclear. Furthermore, cave bats in the karst caves with *S. wulingensis* inhabitants demonstrate clearly seasonal population dynamics, flourishing during the breeding season and declining in winter. Therefore, we suppose that the gene expression profile of *S. wulingensis* is associated with the physiological states. Revealing the gene expression profiles of winter and summer *S. wulingensis* may provide insights into the physiological adaptation of this cave‐dwelling leech to the seasonal dynamics of the bat population in the caves.

The aim of this study was to obtain the transcriptome available for revealing the gene expression profiles of *S. wulingensis* and the molecular basis of the sensory system in this leech adapting to the cave environment. *Sinospelaeobdella wulingensis* samples in a karst cave were collected in winter and summer and applied for RNA sequencing. Gene expression profiles in these samples were analyzed. Candidate Piezos, TRPs, and iGluRs were identified for the first time and inferred to have roles in the sensory system of this troglobite.

## 2 Materials and Methods

### 2.1 Sampling

Nonreproductive *S. wulingensis* were collected from Tangledong cave (28°17′N, 109°39′E), a karst cave in Hunan Province, China, in winter (January 27, 2024) and summer (June 7, 2024). To prevent RNA degradation, leech samples for RNA‐sequencing were frozen in liquid nitrogen and then stored at −70°C. For preparing the RT‐qPCR templates, the oral and caudal suckers, and the main body without suckers were collected from three adults and frozen in liquid nitrogen immediately.

### 2.2 RNA Sequence

The cDNA library construction was delegated to Biomarker Technologies (China) and the RNA sequence was conducted in Illumina NextSeq 500. Raw reads generated from the cDNA library were stringently filtered by removing reads with a sequencing adaptor, > 10% indeterminate bases, or > 50% bases with a quality score of *Q* ≤ 20. Unigenes were generated by assembling clean reads with Trinity software. Gene annotation was conducted by blasting against various databases, including NCBI non‐redundant protein sequences (Nr), NCBI nucleotide sequences (Nt), Protein family (Pfam), euKaryotic Ortholog Groups (KOG), Swiss‐Prot, Kyoto Encyclopedia of Genes and Genomes (KEGG), and Gene Ontology (GO). Gene expression levels were analyzed using the method of fragments per kilobase of transcript sequence per million reads (FPKM). Differentially expressed gene (DEG) analysis was conducted using the false discovery rate (FDR) method. A threshold value of *p*_val_ < 0.05 and an absolute value of log_2_(FoldChange) > 1 was used to identify DEGs.

### 2.3 Identification of Piezo, TRPs, and iGluR Genes

Candidate Piezo, TRP, and iGluR genes in *S. wulingensis* transcriptome were identified by analyzing the gene annotation and were further characterized by sequence analysis. Open Reading Frame Finder (n.d.) (https://www.ncbi.nlm. nih.gov/orffinder/) was used for finding the open reading frames (ORFs) (Sayers et al. 2011). Signal peptide (SP) was predicted in SignalP‐6.0 (Nielsen et al. 2024). Molecular weight (*Mw*) and the theoretical isoelectric point (*pI*) were predicted in Compute pI/Mw web service (Bjellqvist et al. 1994). DeepTMHMM‐1.0 was used for transmembrane domain prediction (Hallgren et al. 2022). CD‐search was applied to identify the conserved domains and protein family attribution of the putative proteins (Wang et al. 2023). Multiple sequence alignment was aligned using Clustal Omega (Madeira et al. 2024). MEGA 11 software was used to construct the phylogenetic trees using the maximum likelihood method (1000 bootstrapping replicates) (Tamura, Stecher, and Kumar 2021). Piezos, TRPs, and iGluRs used in phylogenetic analysis are shown in Tables S1–S3.

### 2.4 Tissue Expression Analysis of Piezo, TRP, and iGluR Genes

Total RNA was extracted using an Animal Total RNA Isolation Kit B518621 (Sangon Biotech, China). RNA concentration and purity were measured in a BioPhotometer D30 (Eppendorf, Germany). First strand cDNA was synthesized from 1 μg of total RNA using EasyScript One‐Step gDNA Removal and cDNA Synthesis SuperMix (Transgen Biotech, China), and then stored at −20°C. RT‐qPCR was conducted by utilizing a QuantStudio 1 Real‐Time PCR System (ThermoFisher, USA). Beta actin (β‐actin) and tubulin alpha chain (α‐tubulin) were used as reference genes. Primers used for RT‐qPCR are shown in Table S4. Each RT‐qPCR reaction was replicated three times. The 2^−ΔΔCt^ method was used for the relative quantification analysis of gene expression. All data were normalized to β‐actin and α‐tubulin expression levels. Gene expression level in the main body was used for calibrating the relative fold changes. Gene expression levels were analyzed using the SPSSAU online service by one‐way analysis of variance (ANOVA) with Tukey's honestly significant difference tests (Tukey 1949; Zou et al. 2025). All data were presented as the mean of triplicates ± standard error (SE).

## 3 Results

### 3.1 Transcriptome Overview

Transcriptome analysis of the winter and summer samples of *S. wulingensis* presents 155.92 million clean reads, including 75.17 million in winter samples and 80.75 million in summer samples, which were composed of 46.4 billion bases (Table S5). The Q30 values of the clean data were 95.01%–95.98%. A total of 29,286 unigenes were obtained by assembling the clean reads. The mean length and N50 of the unigenes are 1386 and 2748 bp, respectively, which indicate the high integrity of the assembly.

**FIGURE 2:**
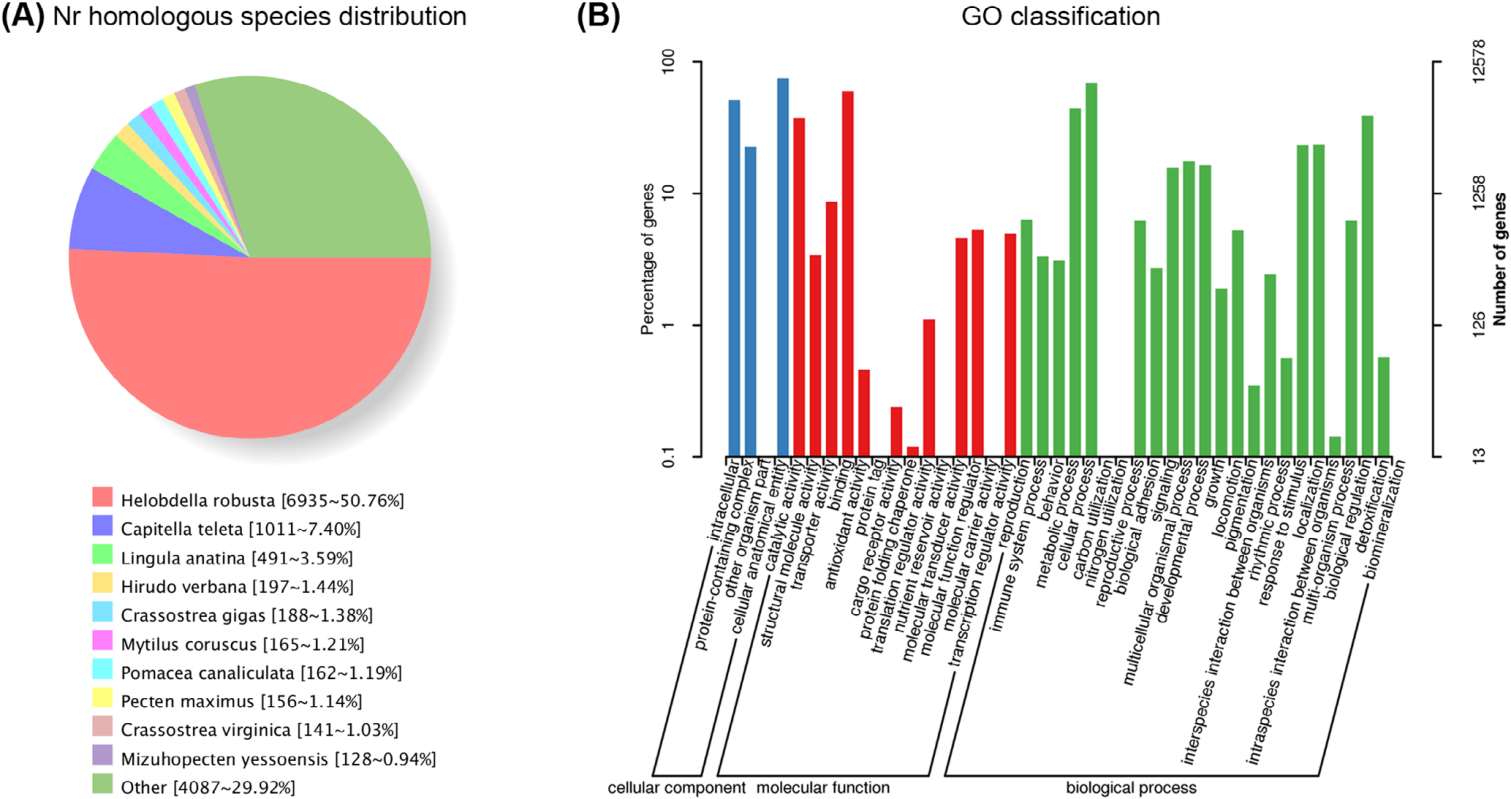
Nr and Go annotation of unigenes. (A) Nr homologous species distribution. Nearly half of the Nr‐annotated unigenes had best hits to the protein sequences in *Helobdella robusta* , followed by some annulata and mollusk species. (B) Histogram description of GO enrichment. The top 42 most enriched GO functional terms are shown.

Functional annotation of the unigenes was conducted in different databases. 50.23% (14,709 unigenes) of the unigenes were annotated in at least one database. A total of 13,661 unigenes are annotated in the Nr database. Nearly half (50.76%, 6935 unigene) of the Nr‐annotated unigenes had best hits to sequences in the leech *Helobdella robusta* , followed by some annulata and mollusk species, such as *Capitella teleta* (7.40%, 1011 unigene), *Lingula anatina* (3.59%, 491 unigene), *Hirudo verbana* (1.44%, 197 unigene), and *Crassostrea gigas* (1.38%, 188) (Figure 2A). In the GO annotation, 12,578 unigenes were assigned to functional terms in three categories: Biological process, Cellular component, and Molecular function (Figure 2B). “Cellular anatomical entity” and “intracellular” in cellular component, “cellular process” and “metabolic process” in biological process, and “binding” in molecular function are the top five most abundant functional terms. Significantly, “response to stimulus” is one of the top five most abundant functional terms in Biological process. 1821 unigenes were mapped to these functional terms, which imply a highly developed sensory system in *S. wulingensis* .

### 3.2 Differentially Expressed Gene Analysis

**FIGURE 3:**
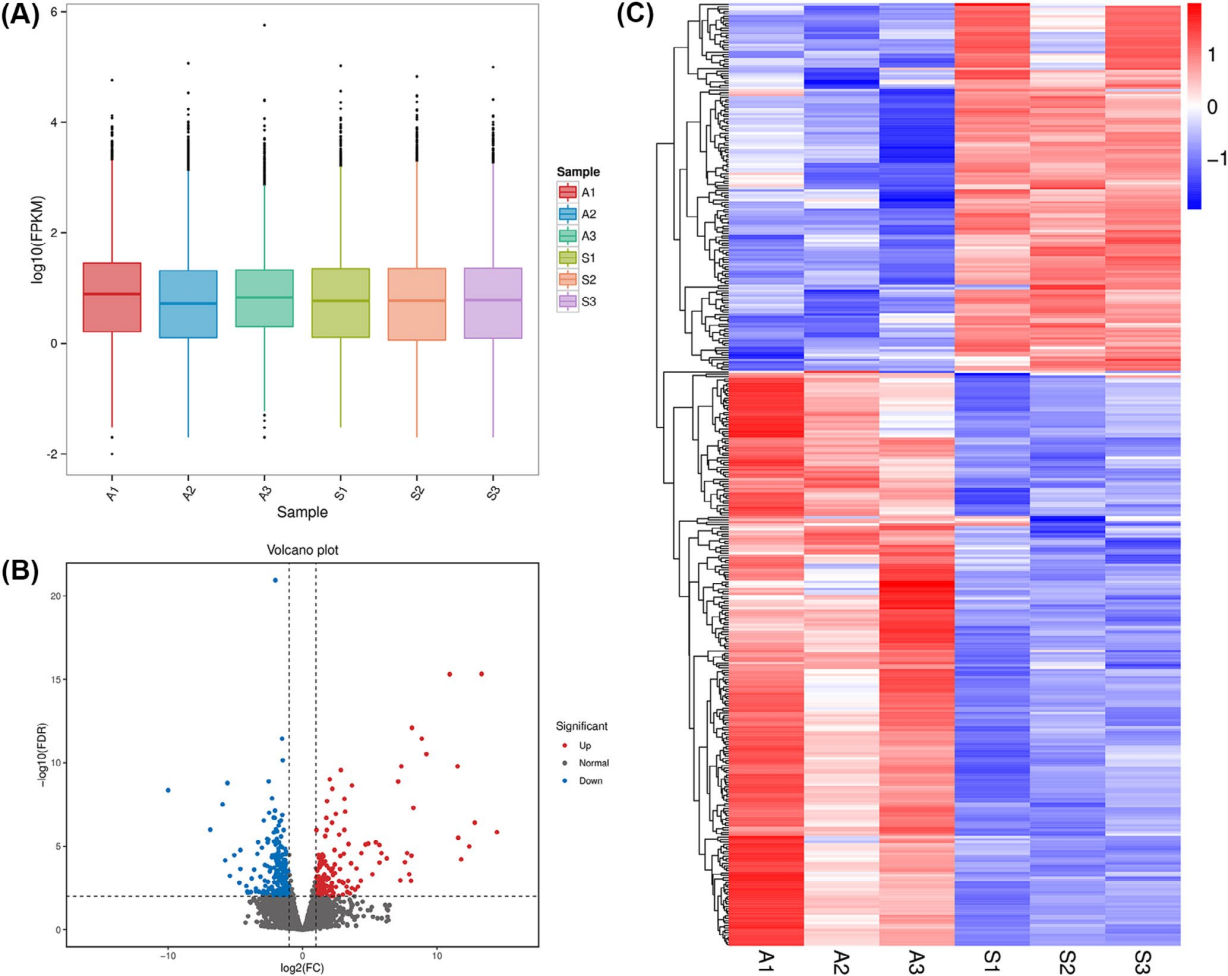
Gene expression analysis based on FPKM. (A) FPKM value statistic of the samples. A1, A2, and A3 are samples collected in winter; S1, S2, and S3 are samples collected in summer. (B) Volcano plot of gene expression analysis. 395 genes were differentially expressed between winter and summer samples. (C) Heat map of DEGs. Relative expression levels of genes in different samples are indicated by a color scale ranging from red to blue. Red boxes represent highly expressed genes. Blue boxes represent lowly expressed genes.

Gene expression profiles in *S. wulingensis* were revealed by analyzing the FPKM values, and the log_10_(FPKM) of the genes in different samples is generally in the range of −2 to 4 (Figure 3A). DEG analysis showed that 395 genes were differentially expressed in comparison with winter and summer *S. wulingensis* samples, including 241 genes upregulated in winter samples and 154 genes upregulated in summer samples (Figure 3B,C).

**FIGURE 4:**
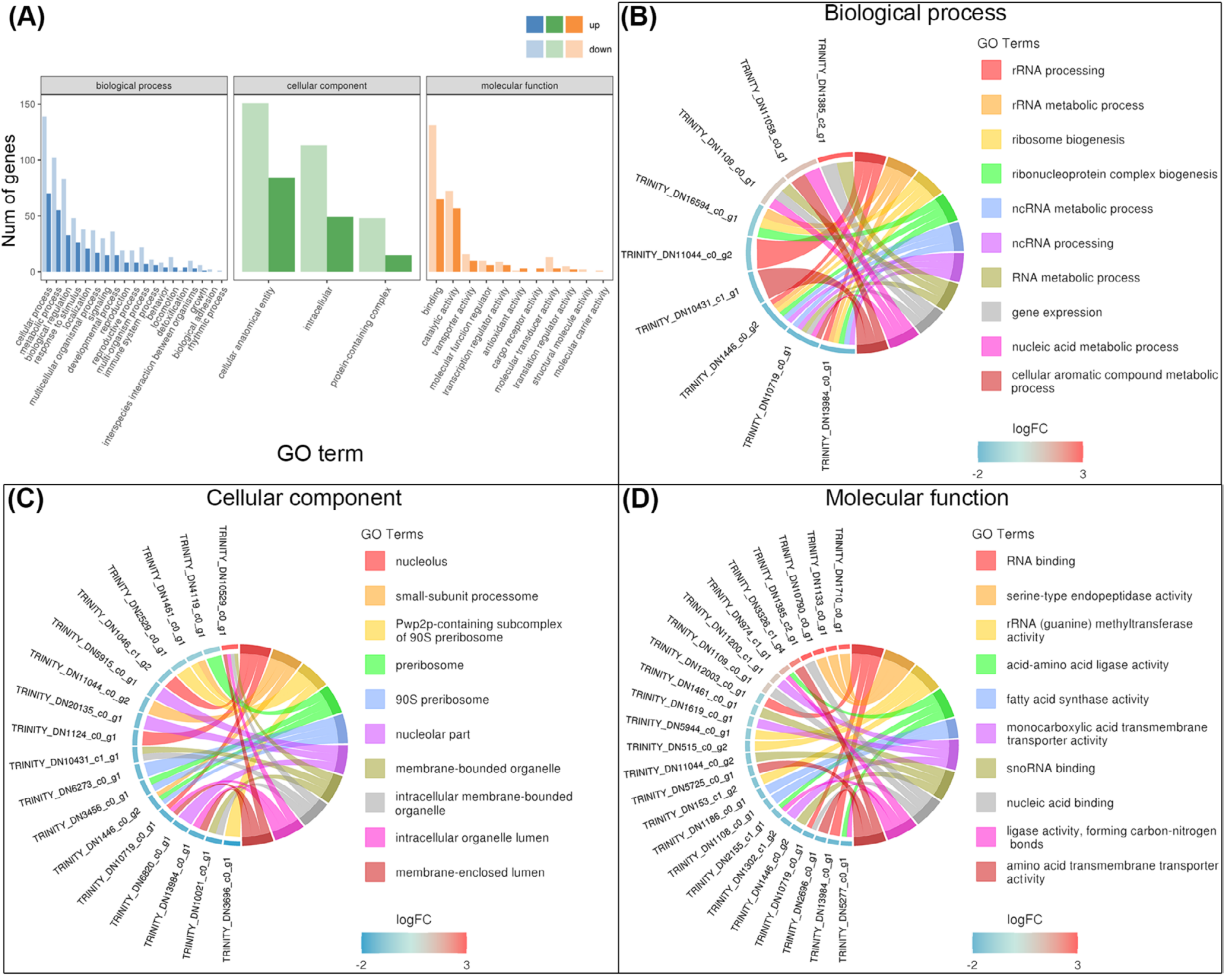
GO enrichment of DEGs. (A) Histogram description of GO enrichment. DEGs match 33 GO functional class in three categories: Biological process, Cellular component, and Molecular function. (B) Top 10 most enriched GO terms in Biological process; these terms are linked with 9 unigenes. (C) Top 10 most enriched GO terms in Cellular component; these terms are linked with 18 unigenes. (D) Top 10 most enriched GO terms in Molecular function; these terms are linked with 25 unigenes.

The putative functions of the DEGs were analyzed in the GO database. These DEGs match 33 GO function classes in three categories: Biological process (19 classes), Cellular component (3 classes), and Molecular function (11 classes) (Figure 4A). “Cellular anatomical entity” in cellular component, “catalytic activity” and “binding” in molecular function, and “metabolic process” and “cellular process” in biological process are the top five most abundant GO classes. The top 10 most significant GO functional terms in the Biological process are linked with 9 genes, and those in the Cellular component and Molecular function matched 18 and 25 genes, respectively (Figure 4B–D). These GO terms are mainly involved in RNA metabolism, gene expression, organelle function, behavior, substance metabolism and transport, response to stimulus, and some other functions.

### 3.3 Identification of Putative Sensory Ion Channels/Receptors

In order to reveal the sensory adaptation of *S. wulingensis* to a hanging living style in the karst caves, putative mechanosensitive ion channels, two Piezos (*SwPiezo1* and *SwPiezo2*) and eight SwTRPs (*SwTRP1‐8*), and candidate chemosensory receptors, six GluRs (SwiGluR1‐6), were identified by analyzing the transcriptome data. *SwPiezo1*, *SwTRP1*, *SwTRP2*, *SwTRP4*, *SwTRP5*, *SwiGluR2*, *SwiGluR4*, *SwiGluR5*, and *SwiGluR6* contain the complete ORF. *SwPiezo2*, *SwTRP3*, *SwTRP6*, *SwTRP7*, *SwTRP8*, *SwiGluR1*, and *SwiGluR3* had partial ORFs. The ORFs of these genes were deposited in the GenBank database. The gene length and predicted *pI* and *MW* are shown in Table S6.

#### 3.3.1 Characterization of Piezo‐Type Mechanosensitive Ion Channels

**FIGURE 5:**
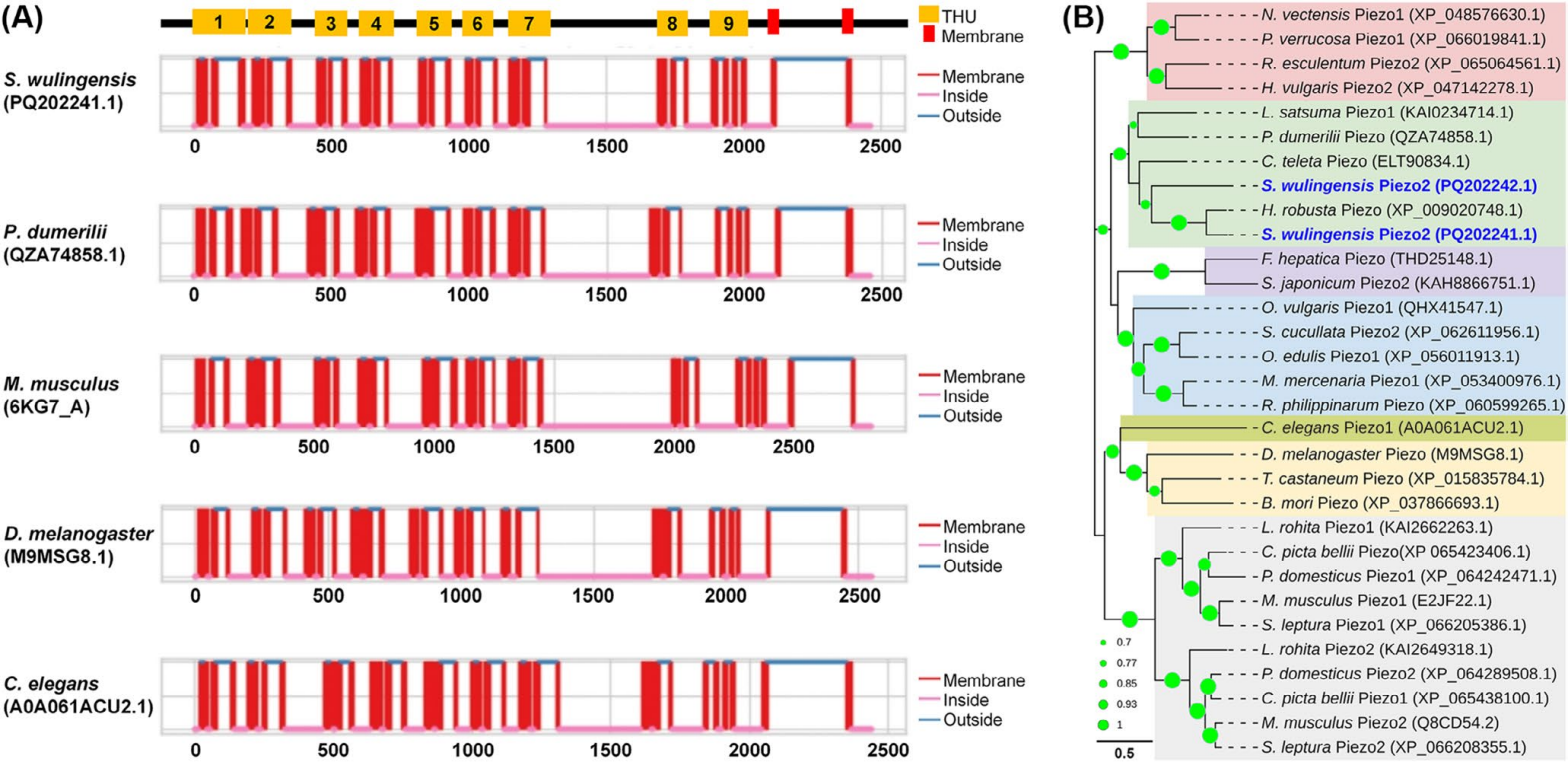
Structural and phylogenetic analyses of SwPiezos and some known Piezos in other species. (A) Protein structure features of SwPiezos and Piezos in some other species, SwPiezo1 share similar TM pattern with other Piezos. (B) Phylogenetic tree constructed by SwPiezos and Piezo form some invertebrates and vertebrates. SwPiezo1 neighbors the clade of *Helobdella robusta* Piezo. SwPiezo2 is located on the edge of Annelida branches. The GenBank accession numbers are listed in the brackets after the Piezos names. Bootstrap values lower than 50% are not shown.

SwPiezo1 and SwPiezo2 contain the conserved Piezo domains “PIEZO” (pfam15917) and “Piezo_RRas_bdg” (pfam12166). The protein sequence identity between SwPiezo1 and SwPiezo2 is 36.97%. Transmembrane analysis indicated that SwPiezo1 is a large transmembrane protein with 38 transmembrane helices (TMs). The former 36 TMs are predicted to form nine transmembrane helical units (THUs). Each THU contains four contiguous TMs (Figure 5A). A conserved extracellular domain of the Piezo family was found at the C‐terminus (Phe2120 to Thr2371) of PxanPiezo, which is the putative domain involved in the formation of a nonspecific cation channel together with TM37 and TM38.

SwPiezo1 and SwPiezo2 share a close relationship with Piezos in other animals. The protein sequence identities of SwPiezo1 with Piezos are 68.85% for *H. robusta* (XP_009020748.1), 40.35% for *Platynereis dumerilii* (QZA74858.1), 37.36% for *Mus musculus* (6KG7_A), 35.03% for *Drosophila melanogaster* (M9MSG8) and 29.58% for *Caenorhabditis elegans* (A0A061ACU2.1). That of SwPiezo2 with Piezos are 39.33% for *P. dumerilii* (QZA74858.1), 36.61% for *H. robusta* (XP_009020748.1), 36.44% for *M. musculus* (6KG7_A), 31.57% for *D. melanogaster* (M9MSG8) and 29.58% for *C. elegans* (A0A061ACU2.1). In the phylogenetic tree, Piezos are clustered into groups by the phylogeny of the animal phyla (Figure 5B). Piezo1 and Piezo2 in Chordates and Cnidarians are grouped into two branches. The annelidan Piezo group neighbors the molluscan Piezo group. SwPiezo1 and SwPiezo2 are in the group of Annelida Piezos. Both of them neighbor the Piezo from an aquatic leech *H. robusta* .

#### 3.3.2 Characterization of Transient Receptor Potential Channels

**FIGURE 6:**
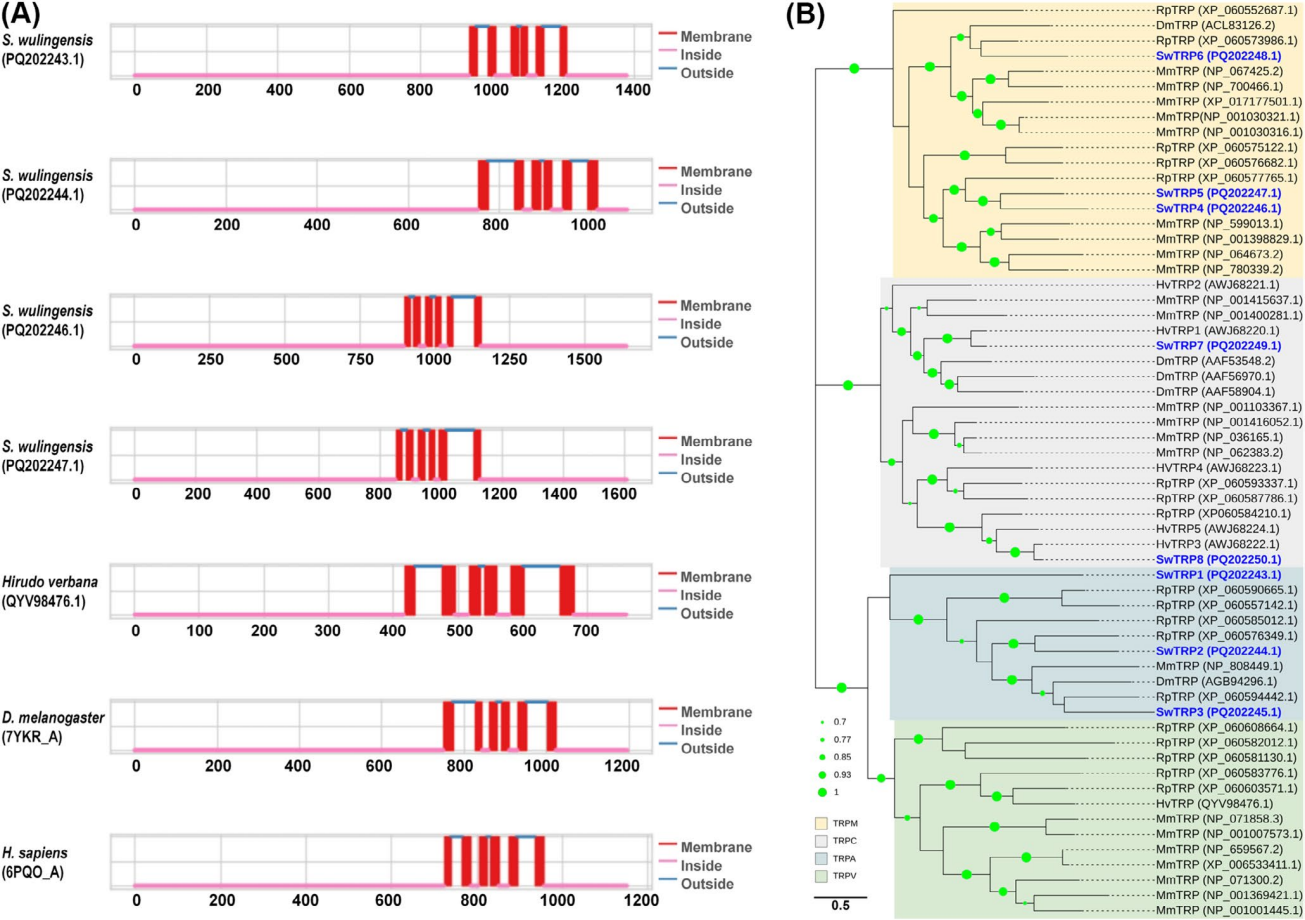
Structural and phylogenetic analyses of SwTRPs and some known TRPs in other species. (A) Protein structure features of SwTRPs and TRPs in some other species, SwTRPs contain six TMs, and the N‐ and C‐termini are located intracellularly. (B) Phylogenetic tree constructed by SwTRPs and TRP from invertebrates and vertebrates. TRPs are clustered into branches by the protein classification and assigned to four subfamilies. The GenBank accession numbers are listed in the brackets after the TRP names. Bootstrap values lower than 50% are not shown.

The identified TRPs in *S. wulingensis* are members of three different TRP subfamilies, including TRPA (SwTRP1, SwTRP2, and SwTRP3), TRPM (SwTRP4, SwTRP5, and SwTRP6), and TRPC (SwTRP7 and SwTRP8). SwTRPs are highly diverse in protein sequence. Pairwise comparison showed that the identity among these SwTRPs ranged from no significant identity to 38.57%. Protein structure analysis indicated that SwTRP1, SwTRP2, SwTRP4, and SwTRP5 are transmembrane proteins containing six TMs (Figure 6A). Both the C‐ and N‐termini of these proteins are located intracellularly. In addition, at least six transmembrane domains were also found in the partial SwTRP3, SwTRP6, SwTRP7, and SwTRP8. Ankyrin repeat sequences were found in the intracellular N‐terminal structural domains of SwTRP1, SwTRP2, and SwTRP3. SwTRP4 and SwTRP5 specifically contain a NUDIX (nucleoside diphosphate‐linked moiety X) motif at the C‐terminal cytoplasmic domain.

BlastX showed that each SwTRP has a best hit with sequences from species in Annelida and Mollusca with identities ranging from 36.64% to 91.20% (Table S6). The phylogenetic tree was constructed with TRPs from *S. wulingensis* , *H. verbana*, *Ruditapes philippinarum*, *D. melanogaster* , and *M. musculus* (Figure 6B). These TRPs are clustered into branches by the protein classification and assigned to four subfamilies. SwiTRP1, SwiTRP2, and SwiTRP3 are located in the branch of TRPA; SwiTRP1 is located on the edge of the TRPA branch; SwiTRP2 and SwiTRP3 are orthologous to TRPs from *R. philippinarum* and *D. melanogaster* , respectively. SwTRP4, SwTRP5, and SwTRP6 belong to the group of TRPM. SwTRP7 and SwTRP8 are in the branch of TRPC; each of them meets an ortholog from *H. verbana*, respectively.

#### 3.3.3 Characterization of Ionotropic Glutamate Receptors

**FIGURE 7:**
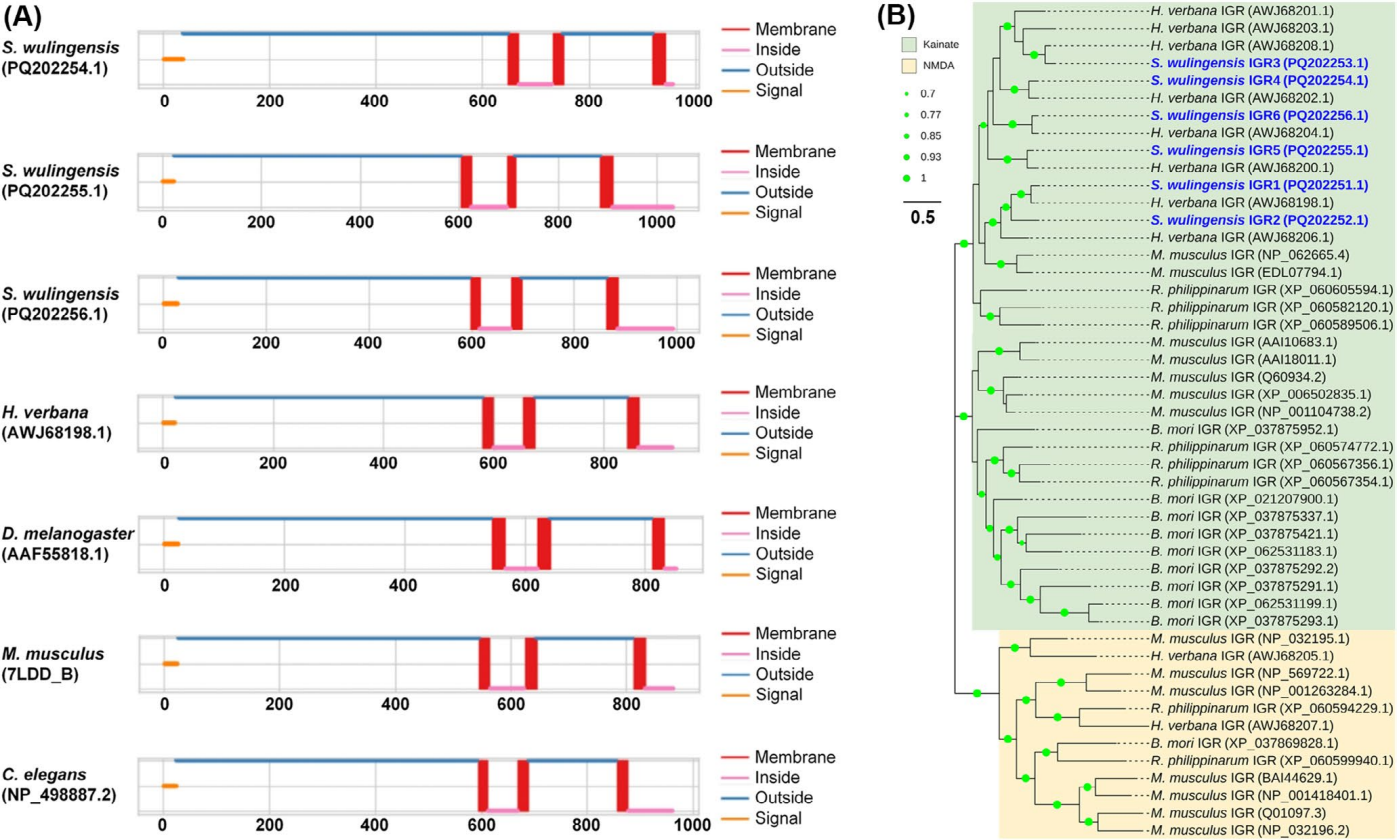
Structural and phylogenetic analyses of SwiGluRs and some known iGluRs in other species. (A) Protein structure features of SwiGluRs and iGluRs in some other species. SwiGluRs are transmembrane protein with extracellular N‐terminal and intracellular C‐terminal; three TMs and a N‐terminal signal sequence were found in SwiGluRs. (B) Phylogenetic tree constructed by SwiGluRs and iGluRs form invertebrates and vertebrates. These GluRs are clustered into two subfamilies. SwiGluRs are located in the group of kainate iGluRs. The GenBank accession numbers are listed in the brackets after the GluRs names. Bootstrap values lower than 50% are not shown.

The iGluRs (SwiGluR1‐6) identified in *S. wulingensis* are members of kainate receptor‐like iGluRs with conserved regions: receptor family ligand binding region (pfam01094) and ligand‐gated ion channel (pfam00060). Pairwise comparison showed that these SwiGluRs are conserved in protein sequence, and the identities among them are 35.50%–61.89%. Prediction of TMs indicated that SwiGluR4, SwiGluR5, and SwiGluR6 are transmembrane proteins with N‐terminal signal sequences (Figure 7A). Each of them contains three TMs in the C‐terminal domain. In addition, putative TMs are also found in the partial SwiGluR1, SwiGluR2, and SwiGluR3. In SwiGluR4, SwiGluR5, and SwiGluR6, the N‐terminal domain and the region between TM2 and TM3 are predicted to form two extracellular domains involved in ligand binding.

BlastX searching showed that each of the SwiGluRs has a best hit matching to iGluRs in the leech *H. verbana* and the identities range from 81.69% to 87.91% (Table S6). In addition, SwiGluRs also share a close relationship with kainate iGluRs in other Phyla. The identities of SwiGluRs with iGluRs in *M. musculus* (7LDD_B) and *C. elegans* (NP_498887.2) are 39.85%–59.06% and 32.18%–48.71%, respectively. In the phylogenetic tree constructed by coelenterate, annelidan, and molluscan iGluRs, the kainate receptor‐like iGluRs cluster together (Figure 7B). SwiGluRs are located in the group of kainate receptor‐like iGluRs. Most of the SwiGluRs, except SwiGluR2, match the orthologs from *H. verbana* in the phylogenetic tree.

#### 3.3.4 Expression Pattern of SwPiezo and SwiGluRs

**FIGURE 8:**
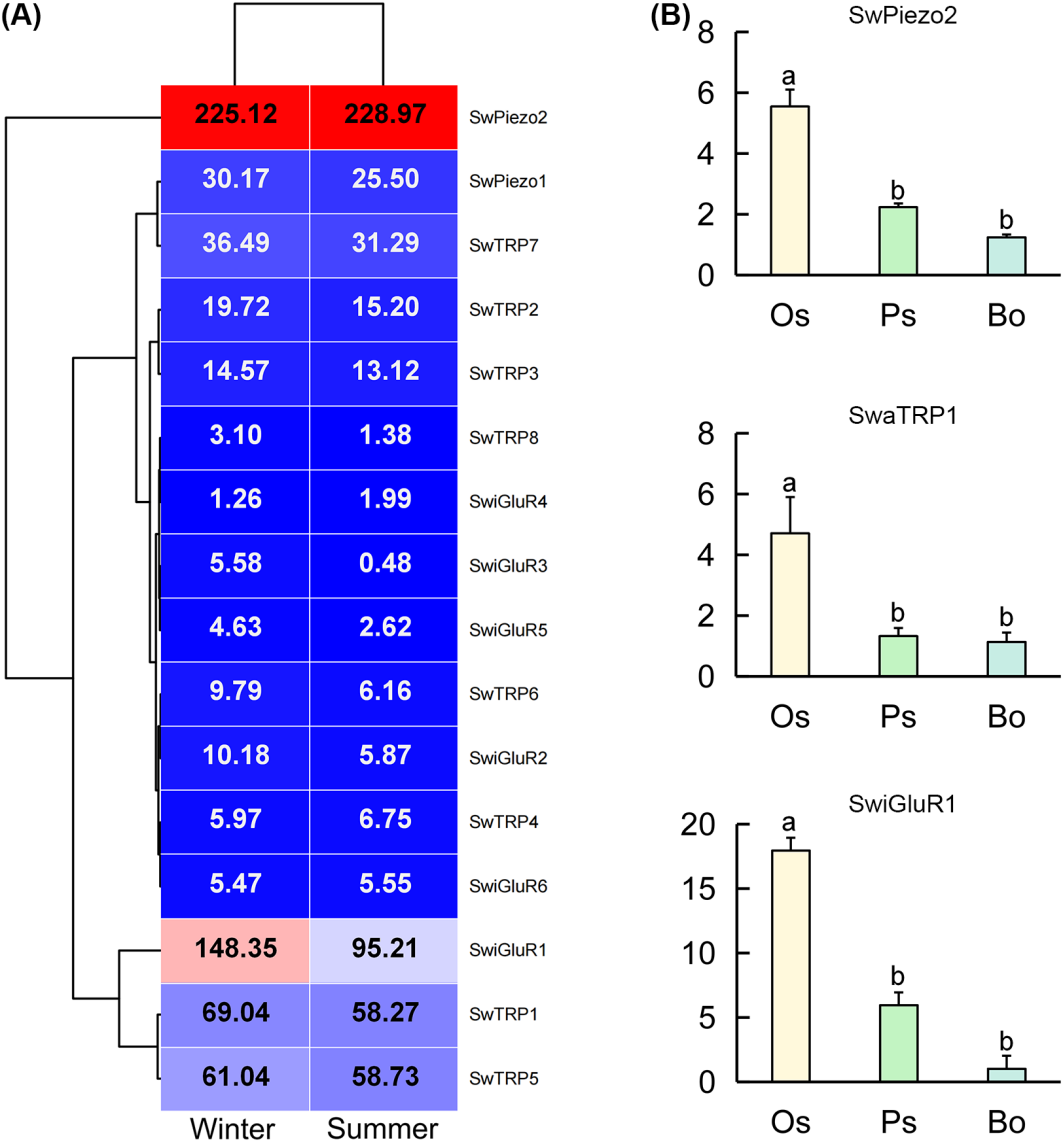
Expression analysis of *Sinospelaeobdella wulingensis* sensory genes. (A) Heat map of *S. wulingensis* sensory genes. Cluster analyses were based on FPKM. Each column represents a sample and each row represents a gene. Red boxes represent highly expressed genes. Blue boxes represent lowly expressed genes. FPKM values are shown in the boxes. (B) Gene expression of SwPiezo2, SwTRP1, and SwiGluR1. Significant differences are marked with letters (*p* < 0.05, one‐way ANOVA). All values are mean ± SE.

In the DEG analysis based on FPKM values, gene expression of SwPiezos, SwTRPs, and SwiGluRs did not present a significant difference between the samples collected in winter and summer (Figure 8A). Most of the SwPiezos, SwTRPs, and SwiGluRs, except SwiGluR3, are expressed in the winter and summer samples with FPKM > 1. Moreover, SwPiezo1, SwPiezo2, SwTRP1, SwTRP2, SwTRP3, SwTRP5, SwTRP7, and SwiGluR1 are abundantly expressed in both samples with FPKM > 10. Tissue distribution of SwPiezo2, SwTRP1, and SwiGluR1 in the suckers and main body was further determined by RT‐qPCR (Figure 8B). The results showed that SwPiezo2, SwTRP1, and SwiGluR1 are primarily expressed in the suckers. Their expression levels in the oral sucker are significantly higher than those in the caudal sucker. While their expression in the caudal sucker is also higher than that in the main body, it is without a significant difference.

## 4 Discussion

The cave‐dwelling land leech *S. wulingensis* is a temporary parasite of cave bats in the karst caves. Fade of surface pigment and extensive oral and caudal suckers were observed in this leech, indicating the extreme adaptation of this troglobite to the hanging living style in the karst caves (Huang, Liu, et al. 2019). Mechanosensory and chemosensory systems have important roles in locomotion coordination and chemotactic responses, which are the biological base for animal behavior (Daghfous et al. 2012; Zhang et al. 2015). In this study, we conducted a de novo transcriptome analysis of *S. wulingensis* and identified for the first time presenting putative mechanosensory and chemosensory genes in leeches, including two SwPiezos, eight SwTRPs, and six SwiGluRs. 29,286 unigenes were obtained by assembling the clean reads, and only 395 genes are differentially expressed between winter and summer samples. The similar gene expression profiles in winter and summer samples suggested the stable physiological status of *S. wulingensis* in the cave environment different from the surface world. SwPiezos, SwTRPs, and SwiGluRs are transmembrane proteins sharing conserved structural features in the respective protein families. SwPiezo1, SwPiezo2, SwTRP1, SwTRP2, SwTRP3, SwTRP5, SwTRP7, and SwiGluR1 are abundantly expressed in both winter and summer samples. Expression levels of SwPiezo2, SwTRP1, and SwiGluR1 in the oral sucker are significantly higher than those in the caudal sucker and body. These results suggest that mechanosensory and chemosensory genes identified in this troglobite may play roles in locomotion coordination and prey perception in the karst caves.

Animal Piezos are sensory proteins functionally involved in the perception of mechanical stimulation (Kim et al. 2012). These proteins are very large, evolutionarily conserved transmembrane proteins, and generally function as homotrimers that are activated by physical force and allow cations to permeate cells (Lee et al. 2024). A recent study in mice showed that Piezo1 and Piezo2 are found in the hair cells of the inner ear and may play roles in hearing and balance (Lee et al. 2024). Furthermore, Piezo1 channels in mice could be activated by ultrasound, initiating Ca^2+^ influx, which implied the potential role of Piezo channels in ultrasound reception (Qiu et al. 2019). Piezos are also widely identified in invertebrates, such as *D. melanogaster* and *C. elegans* , and have been confirmed to be involved in mechanotransduction (Kim et al. 2012; Millet et al. 2022). In this study, SwPiezo1 in the cave‐dwelling leech *S. wulingensis* shares high sequence identity and a conserved TM distribution profile with Piezos in other invertebrates and vertebrates. Furthermore, animal Piezos were assigned to different groups by the phylogeny of the animal phyla in the phylogenetic tree. These results imply that animal Piezos may be conserved in protein sequence and structural features and may play conserved roles in animals from different phyla. In *C. elegans* , *D. melanogaster*, and *M. musculus* , Piezos were functionally involved in cellular and tissue mechanosensitivity by converting mechanical stimuli into bioelectrical signals (Zhang et al. 2023). Expression analysis showed that SwPiezo1 and SwPiezo2 were highly expressed in *S. wulingensis* and SwPiezo2 was primarily expressed in the suckers. Similar to the other land leeches feeding on the blood of vertebrate animals, *S. wulingensis* can move in a geometer style by using the oral and caudal suckers as locomotive organs. Oral and caudal suckers in *S. wulingensis* are prominent, and the caudal sucker diameter is greater than the maximum body breadth, suggesting a morphological adaptation to the hanging living style in the caves (Huang, Liu, et al. 2019). Therefore, we suggest that SwPiezos are candidate mechanical ion channels in *S. wulingensis* and may play important roles in the locomotion coordination of this land leech that has a unique hanging living style, attaching to the roof and wells of karst caves.

TRPs are multimodal ion channels that act as sensors of chemical and physical stimuli such as light, sound, touch, pheromones, and tissue damage (Kindt et al. 2007; Eijkelkamp, Quick, and Wood 2013). In the present study, eight TRPs were identified in *S. wulingensis* . Phylogenetic analysis showed that TRPs from different animal phyla were grouped into branches according to protein subfamilies, and SwTRPs were assigned to three subfamilies. These results implied that TRPs in *S. wulingensis* are a multigene family encoding TRPs belonging to different subfamilies. TRPs are transmembrane proteins containing six transmembrane domains (S1–S6) and forming functional channels as homo‐/hetero‐tetramers. Ankyrin repeats are found in the intracellular N‐terminal structural domains of TRPA, TRPV, TRPC, and TRPN channels (Eijkelkamp, Quick, and Wood 2013; Jin et al. 2017). In animal tissues, ankyrin repeat domains can serve as sensors for different stimuli, such as heat, cold, pressure, pH, and cellular damage (Zhang et al. 2023). In *Drosophila*, a TRPN channel with 29 ankyrin repeats acted as mechanosensory channels (Zhang et al. 2015). Ankyrin repeats in this TRPN are located intracellularly and convey mechanical force from the microtubule cytoskeleton to gate the channels (Liang and Howard 2017). In *S. wulingensis* , SwTRP1, SwTRP2, and SwTRP3 are members of TRPA and contain ankyrin repeat sequences in the N‐terminal structural domains and may play roles in signal transduction from the microtubule cytoskeleton force into electrical signal through the ankyrin repeats. In addition, SwTRP4 and SwTRP5 are members of TRPM and contain a C‐terminal NUDIX motif which is also known as an ADP‐ribose pyrophosphatase domain, specifically catalyzing the hydrolysis of ADP‐ribose to AMP and ribose‐5‐P (Shen et al. 2003). In mammals and insects, TRPM are found in the sensory neurons and smooth muscle cells and play important roles in mechanosensitivity, such as touch, stretch, osmolarity, and shear stress (Eijkelkamp, Quick, and Wood 2013). Taken together, we suggest that SwTRPs are candidate mechanosensory ion channels that may play important roles in response to the mechanical stimuli in the cave environment.

The iGluRs in animals are a highly conserved family of ligand‐gated ion channels found in a wide range of eumetazoa animals (Hansen et al. 2021). In the glutamatergic neuron system, iGluRs are receptors of the neurotransmitter glutamate and play a crucial role in nerve signal transduction (Fossati and Charrier 2021). These iGluRs mediate most excitatory neurotransmission in the central nervous system and are implicated in nearly all aspects of nervous system development and function (Yelshanskaya, Li, and Sobolevsky 2014). Moreover, iGluRs also represent an important signaling mechanism in response to external chemical signals by binding extracellular glutamate and related ligands (Croset et al. 2010; Núñez‐Acuña et al. 2014). Animal iGluRs were initially divided into different classes mainly based on pharmacological properties: AMPA receptors, kainate receptors, NMDA receptors, and GluD receptors (Granger et al. 2011; Burada, Vinnakota, and Kumar 2020). This classification is also in accordance with the sequence identity of iGluRs (Croset et al. 2010). In this study, we identified six iGluRs in *S. wulingensis* . Phylogenetic analysis showed that these SwiGluRs are members of kainate‐like receptors and share a close relationship with the aquatic blood‐sucking leech *H. verbana*, in which transcriptome analysis presented 11 iGluRs, including nine “kainate‐like” and two “NMDA‐like” receptors (Northcutt et al. 2018). These results implied that the leech GluRs are highly expanded in the kainate‐like subfamily in comparison with the other iGluR subfamilies. Furthermore, credible evidence in the literature showed that iGluRs expressed in the sensory tissues are highly associated with chemosensory perception. In the marine echinoderms, iGluR genes were found in tentacles and were inferred to have a chemosensory role (Sania et al. 2021). The variant subfamily of iGluRs, ionotropic receptors (IRs), in insects were characterized as a large olfactory receptor family, playing important roles in detecting the chemosensory signals in the environment. Our findings showed that SwiGluRs share conserved structural features with iGluRs in other invertebrates and vertebrates. SwiGluR1 is primarily expressed in the oral sucker, which is a feeding and sensory center in *S. wulingensis* , containing the mouth and eyes (Huang, Liu, et al. 2019). These findings suggest that SwiGluRs may have roles in chemosensory perception in *S. wulingensis* and indicate that the oral sucker is a sensory center for perceiving chemical signals from the prey in the caves.

Molecular studies on cave animals have greatly contributed to probing for genetic universalities underlying cave adaptation (Friedrich et al. 2011; Huang, Titus, et al. 2019). Revealing the gene expression profiles in organisms could provide insights into the physiological mechanisms mediating adaptation to habitats (Passow et al. 2017). In the karst caves, *S. wulingensis* fed on the blood of bats and presented similar gene expression profiles in the winter and summer samples. Only 395 of 29,286 unigenes were differentially expressed in different samples and matched 33 GO terms, such as “Cellular anatomical entity,” “catalytic activity,” and “binding.” In contrast, starvation‐induced changes in the transcriptome of salivary glands in the leech *Hirudo nipponia* Whitman presented 2650 DEGs that were involved in more than 70 functional terms (Cai et al. 2024). *Sinospelaeobdella wulingensis* generally inhabits deep cave sites with low air mobility, high humidity, a stable microclimate, and long‐term darkness (Huang, Liu, et al. 2019). These sites are also suitable for cave bats' inhabitation and hibernation. Vanderwolf and McAkpine (2021) studied the microclimate of cave bat hibernacula in North America and found that cave bats hibernated at sites with high relative humidity and that the roosting temperatures were more stable than the aboveground temperatures. In the south of China, the bat roosting temperatures in winter and summer are 18.94°C ± 1.35°C and 23.06°C ± 1.26°C, respectively, and the roosting humidity in winter and summer is 79.00 ± 4.66 and 83.27 ± 3.39, respectively (Guo et al. 2022). In Hunan Province, *S. wulingensis* shares the microhabitat with some cave bats (Huang, Liu, et al. 2019). A stable microclimate in different seasons may provide a stable living environment for the cave leech. Inhabiting and hibernating bats could provide a continuous food resource. We suggest that similar gene expression profiles in the winter and summer *S. wulingensis* samples imply a stable physiological status of this troglobite in a cave environment.

In conclusion, we conducted a transcriptome analysis on the cave‐dwelling leech *S. wulingensis* and presented 29,286 unigenes; only 395 genes are differentially expressed in winter and summer samples. SwPiezos, SwTRPs, and SwiGluRs in *S. wulingensis* are transmembrane proteins sharing conserved structural features in respective protein families. Two SwPiezos, five SwTRPs, and one SwiGluR are abundantly expressed in both winter and summer samples; SwPiezo2, SwTRP1, and SwiGluR1 share similar tissue distribution patterns and are primarily expressed in the oral sucker. These results suggest that SwPiezos, SwTRPs, and SwiGluRs are candidate sensory proteins that may have roles in locomotion coordination and host perception. Enrichment of sensory genes in the oral sucker indicates the important role of this tissue in response to environmental stimuli. Similar gene expression profiles in winter and summer *S. wulingensis* samples imply a stable physiological status of this troglobite in the cave environment.

## Author Contributions

**Xi Wen:** software (equal), writing – original draft (equal). **Haiyang Xiang:** writing – original draft (equal). **Mengqing Zhang:** writing – original draft (equal). **Aoran Yan:** writing – original draft (equal). **Dongqing Xiang:** writing – original draft (equal). **Jie Zou:** writing – original draft (equal). **Yue Zhang:** writing – original draft (equal). **Xinglong Huang:** conceptualization (equal), methodology (equal), writing – review and editing (equal). **Zhixiao Liu:** writing – original draft (equal).

## Conflicts of Interest

The authors declare no conflicts of interest.

## Benefit‐Sharing Section

Benefits from this research accrue from the sharing of our data and results on public databases, as described above.

## Supporting information


Appendix S1.

## Data Availability

All raw data and sample metadata generated for this project are stored in the NCBI Short Read Archive (SRA) under project PRJNA1136657 (https://www.ncbi.nlm.nih.gov/bioproject/PRJNA1136657).
