# Supplementary material for: Molecular Insights Into the Sensory Adaption of the Cave‐Dwelling Leech Sinospelaeobdella wulingensis to the Karst Cave Environment
Source: Ecol Evol. 2025 Jan 18;15(1):e70877. doi: 10.1002/ece3.70877 (PMC11748453; doi:10.1002/ece3.70877)
Supplement: Supplementary file 1 — Appendix S1. [file ECE3-15-e70877-s001.doc]

**Table S1. Piezos used in in phylogenetic analysis**

| Phylum | Species | Protein | Accession NO. |
| --- | --- | --- | --- |
| Cnidaria | *Nematostella vectensis* | piezo-type mechanosensitive ion channel component 1 | XP_048576630.1 |
|  | *Pocillopora verrucosa* | piezo-type mechanosensitive ion channel component 1 | XP_066019841.1 |
|  | *Rhopilema esculentum* | piezo-type mechanosensitive ion channel component 2-like | XP_065064561.1 |
|  | *Hydra vulgaris* | piezo-type mechanosensitive ion channel component 2 | XP_047142278.1 |
| Annelida | *Sinospelaeobdella wulingensis* | Piezo-type mechanosensitive ion channel component 1 | PQ202241.1 |
|  | *Sinospelaeobdella wulingensis* | Piezo-type mechanosensitive ion channel component 2 | PQ202242.1 |
|  | *Helobdella robusta* | hypothetical protein HELRODRAFT 192356 | XP_009020748.1 |
|  | *Capitella teleta* | hypothetical protein CAPTEDRAFT 219762 | ELT90834.1 |
|  | *Platynereis dumerilii* | piezo | QZA74858.1 |
|  | *Lamellibrachia satsuma* | Piezo-type mechanosensitive ion channel component 1 | KAI0234714.1 |
| Platyhelminthes | *Schistosoma japonicum* | Piezo-type mechanosensitive ion channel component 2 | KAH8866751.1 |
|  | *Fasciola hepatica* | Piezo-type mechanosensitive ion channel component | THD25148.1 |
| Mollusca | *Octopus vulgaris* | piezo-type mechanosensitive ion channel component 1-like | QHX41547.1 |
|  | *Saccostrea cucullata* | piezo-type mechanosensitive ion channel component 2 | XP_062611956.1 |
|  | *Ostrea edulis* | piezo-type mechanosensitive ion channel component 1-like | XP_056011913.1 |
|  | *Mercenaria mercenaria* | piezo-type mechanosensitive ion channel component 1-like | XP_053400976.1 |
|  | *Ruditapes philippinarum* | piezo-type mechanosensitive ion channel component 2-like | XP_060599265.1 |
| Nematoda | *Caenorhabditis elegans* | Piezo-type mechanosensitive ion channel component 1 | A0A061ACU2.1 |
| Arthropoda | *Drosophila melanogaster* | Piezo-type mechanosensitive ion channel component | M9MSG8.1 |
|  | *Tribolium castaneum* | piezo-type mechanosensitive ion channel component | XP_015835784.1 |
|  | *Bombyx mori* | piezo-type mechanosensitive ion channel component | XP_037866693.1 |
| Chordata | *Labeo rohita* | Piezo-type mechanosensitive ion channel component 1 | KAI2662263.1 |
|  | *Labeo rohita* | Piezo-type mechanosensitive ion channel component 2 | KAI2649318.1 |
|  | *Chrysemys picta bellii* | piezo-type mechanosensitive ion channel component 1 | XP 065423406.1 |
|  | *Chrysemys picta bellii* | piezo-type mechanosensitive ion channel component 2 | XP_065438100.1 |
|  | *Passer domesticus* | piezo-type mechanosensitive ion channel component 1 | XP_064242471.1 |
|  | *Passer domesticus* | piezo-type mechanosensitive ion channel component 2 | XP_064289508.1 |
|  | *Mus musculus* | Piezo-type mechanosensitive ion channel component 1 | E2JF22.1 |
|  | *Mus musculus* | Piezo-type mechanosensitive ion channel component 2 | Q8CD54.2 |
|  | *Saccopteryx leptura* | piezo-type mechanosensitive ion channel component 1 | XP_066205386.1 |
|  | *Saccopteryx leptura* | piezo-type mechanosensitive ion channel component 2 | XP_066208355.1 |

**Table S2. TRPs used in in phylogenetic analysis**

| Phylum | Species | Protein | Accession NO. |
| --- | --- | --- | --- |
| Annelida | *Sinospelaeobdella wulingensis* | transient receptor potential cation channel 6 | PQ202248.1 |
|  | *Sinospelaeobdella wulingensis* | transient receptor potential cation channel 5 | PQ202247.1 |
|  | *Sinospelaeobdella wulingensis* | transient receptor potential cation channel 4 | PQ202246.1 |
|  | *Sinospelaeobdella wulingensis* | transient receptor potential cation channel 7 | PQ202249.1 |
|  | *Sinospelaeobdella wulingensis* | transient receptor potential cation channel 8 | PQ202250.1 |
|  | *Sinospelaeobdella wulingensis* | transient receptor potential cation channel 1 | PQ202243.1 |
|  | *Sinospelaeobdella wulingensis* | transient receptor potential cation channel 2 | PQ202244.1 |
|  | *Sinospelaeobdella wulingensis* | transient receptor potential cation channel 3 | PQ202245.1 |
|  | *Hirudo verbana* | putative transient receptor potential channel 2 partial | AWJ68221.1 |
|  | *Hirudo verbana* | putative transient receptor potential channel 1 | AWJ68220.1 |
|  | *Hirudo verbana* | putative transient receptor potential channel 4 | AWJ68223.1 |
|  | *Hirudo verbana* | putative transient receptor potential channel 5 | AWJ68224.1 |
|  | *Hirudo verbana* | putative transient receptor potential channel 3 | AWJ68222.1 |
|  | *Hirudo verbana* | transient receptor potential cation channel subfamily V | QYV98476.1 |
| Mollusca | *Ruditapes philippinarum* | transient receptor potential cation channel subfamily M member 2-like | XP_060552687.1 |
|  | *Ruditapes philippinarum* | transient receptor potential cation channel subfamily M member 3-like | XP_060573986.1 |
|  | *Ruditapes philippinarum* | transient receptor potential cation channel subfamily M member-like 2 | XP_060575122.1 |
|  | *Ruditapes philippinarum* | transient receptor potential cation channel subfamily M member-like 2 | XP_060576682.1 |
|  | *Ruditapes philippinarum* | transient receptor potential cation channel subfamily M member-like 2 | XP_060577765.1 |
|  | *Ruditapes philippinarum* | short transient receptor potential channel 7-like | XP_060593337.1 |
|  | *Ruditapes philippinarum* | short transient receptor potential channel 7-like | XP_060587786.1 |
|  | *Ruditapes philippinarum* | short transient receptor potential channel 7-like | XP_060584210.1 |
|  | *Ruditapes philippinarum* | transient receptor potential cation channel subfamily A member 1 homolog | XP_060590665.1 |
|  | *Ruditapes philippinarum* | transient receptor potential cation channel subfamily A member 1 homolog partia | XP_060557142.1 |
|  | *Ruditapes philippinarum* | transient receptor potential cation channel subfamily A member 1 homolog | XP_060585012.1 |
|  | *Ruditapes philippinarum* | transient receptor potential cation channel subfamily A member 1-like | XP_060576349.1 |
|  | *Ruditapes philippinarum* | transient receptor potential cation channel subfamily A member 1-like | XP_060594442.1 |
|  | *Ruditapes philippinarum* | transient receptor potential cation channel subfamily V member 6-like | XP_060608664.1 |
|  | *Ruditapes philippinarum* | transient receptor potential cation channel subfamily V member 5-like | XP 060582012.1 |
|  | *Ruditapes philippinarum* | transient receptor potential cation channel subfamily V member 1-like | XP_060581130.1 |
|  | *Ruditapes philippinarum* | transient receptor potential cation channel subfamily V member 6-like | XP_060583776.1 |
|  | *Ruditapes philippinarum* | transient receptor potential cation channel subfamily V member 5-like | XP_060603571.1 |
| Arthropoda | *Drosophila melanogaster* | transient receptor potential cation channel gamma | AAF53548.2 |
|  | *Drosophila melanogaster* | transient receptor potential | AAF56970.1 |
|  | *Drosophila melanogaster* | transient receptor potential-like | AAF58904.1 |
|  | *Drosophila melanogaster* | transient receptor potential cation channel subfamily M | ACL83126.2 |
|  | *Drosophila melanogaster* | transient receptor potential cation channel A1 | AGB94296.1 |
| Chordata | *Mus musculus* | transient receptor potential cation channel subfamily M member 3 | N_001030321.1 |
|  | *Mus musculus* | transient receptor potential cation channel subfamily M member 7 | NP_067425.2 |
|  | *Mus_musculus* | transient receptor potential cation channel subfamily M member 6 | NP_700466.1 |
|  | *Mus musculus* | transient receptor potential cation channel subfamily M member 1 | XP_017177501.1 |
|  | *Mus musculus* | transient receptor potential cation channel subfamily M member 3 | NP_001030316.1 |
|  | *Mus musculus* | transient receptor potential cation channel subfamily M member 8 | NP_599013.1 |
|  | *Mus musculus* | transient receptor potential cation channel subfamily M member 2 | NP_001398829.1 |
|  | *Mus musculus* | short transient receptor potential channel 4 | NP_001415637.1 |
|  | *Mus musculus* | short transient receptor potential channel 1 | NP_001400281.1 |
|  | *Mus musculus* | short transient receptor potential channel 2 | NP_001103367.1 |
|  | *Mus musculus* | short transient receptor potential channel 6 | NP_001416052.1 |
|  | *Mus musculus* | short transient receptor potential channel 7 | NP_036165.1 |
|  | *Mus musculus* | transient receptor potential cation channel subfamily V member | NP_071858.3 |
|  | *Mus musculus* | transient receptor potential cation channel subfamily V member 5 | NP_001007573.1 |
|  | *Mus musculus* | transient receptor potential cation channel subfamily V member 3 | NP_659567.2 |
|  | *Mus musculus* | transient receptor potential cation channel subfamily V member 4 | NP_071300.2_ |
|  | *Mus musculus* | transient receptor potential cation channel subfamily V member 2 | NP_001369421.1 |
|  | *Mus musculus* | transient receptor potential cation channel subfamily V member 1 | NP_001001445.1 |
|  | *Mus musculus* | transient receptor potential cation channel subfamily M member 5 | NP_064673.2 |
|  | *Mus musculus* | transient receptor potential cation channel subfamily M member 4 | NP_780339.2 |
|  | *Mus musculus* | short transient receptor potential channel 3 | NP_062383.2 |
|  | *Mus musculus* | transient receptor potential cation channel subfamily A member 1 | NP_808449.1 |
|  | *Mus musculus* | transient receptor potential cation channel subfamily V member 3 | XP_006533411.1 |

**Table S3. IGluRs used in in phylogenetic analysis**

| Phylum | Species | Protein | Accession NO. |
| --- | --- | --- | --- |
| Annelida | *Sinospelaeobdella wulingensis* | Ionotropic glutamate receptor 3 | PQ202253.1 |
|  | *Sinospelaeobdella wulingensis* | Ionotropic glutamate receptor 4 | PQ202254.1 |
|  | *Sinospelaeobdella wulingensis* | Ionotropic glutamate receptor 6 | PQ202256.1 |
|  | *Sinospelaeobdella wulingensis* | Ionotropic glutamate receptor 5 | PQ202255.1 |
|  | *Sinospelaeobdella wulingensis* | Ionotropic glutamate receptor 1 | PQ202251.1 |
|  | *Sinospelaeobdella wulingensis* | Ionotropic glutamate receptor 2 | PQ202252.1 |
|  | *Hirudo verbana* | Ionotropic glutamate receptor kainate-like 4 | AWJ68201.1 |
|  | *Hirudo verbana* | Ionotropic glutamate receptor kainate-like 6 | AWJ68203.1 |
|  | *Hirudo verbana* | Ionotropic glutamate receptor kainate-like 9 | AWJ68208.1 |
|  | *Hirudo verbana* | Ionotropic glutamate receptor kainate-like 5 | AWJ68202.1 |
|  | *Hirudo verbana* | Ionotropic glutamate receptor kainate-like 7 | AWJ68204.1 |
|  | *Hirudo verbana* | Ionotropic glutamate receptor kainate-like 3 | AWJ68200.1 |
|  | *Hirudo verbana* | Putative ionotropic glutamate receptor kainate-like 1 | AWJ68198.1 |
|  | *Hirudo verbana* | Ionotropic glutamate receptor kainate-like 8 partia | AWJ68206.1 l |
|  | *Hirudo verbana* | Ionotropic glutamate receptor NMDA-like 1 | AWJ68205.1 |
|  | *Hirudo verbana* | Ionotropic glutamate receptor NMDA-like 2 | AWJ68207.1 |
| Mollusca | *Ruditapes philippinarum* | Glutamate receptor 2-like | XP_060605594.1 |
|  | *Ruditapes philippinarum* | Glutamate receptor 1-like | XP_060582120.1 |
|  | *Ruditapes philippinarum* | Glutamate receptor 4-like | XP_060589506.1 |
|  | *Ruditapes philippinarum* | Glutamate receptor ionotropic kainate 2-like | XP_060574772.1 |
|  | *Ruditapes philippinarum* | Glutamate receptor ionotropic kainate 2-like | XP_060567356.1 |
|  | *Ruditapes philippinarum* | Glutamate receptor ionotropic kainate 1-like | XP_060567354.1 |
|  | *Ruditapes philippinarum* | Glutamate receptor ionotropic NMDA 3A-like partial | XP_060594229.1 |
|  | *Ruditapes philippinarum* | Glutamate receptor ionotropic NMDA 2B-like | XP_060599940.1 |
| Arthropoda | *Bombyx mori* | Glutamate receptor ionotropic kainate 2 | XP_037875952.1 |
|  | *Bombyx mori* | Glutamate receptor ionotropic kainate 2 | XP_021207900.1 |
|  | *Bombyx mori* | Glutamate receptor ionotropic kainate 2 | XP_037875337.1 |
|  | *Bombyx mori* | Glutamate receptor ionotropic kainate 2 | XP_037875421.1 |
|  | *Bombyx mori* | Glutamate receptor ionotropic kainate 3 | XP_062531183.1 |
|  | *Bombyx mori* | Glutamate receptor ionotropic kainate 2 | XP_037875292.2 |
|  | *Bombyx mori* | Glutamate receptor ionotropic kainate 2 | XP_037875291.1 |
|  | *Bombyx mori* | Glutamate receptor ionotropic kainate 2 | XP_062531199.1 |
|  | *Bombyx mori* | Glutamate receptor ionotropic kainate 2 | XP_037875293.1 |
|  | *Bombyx mori* | Glutamate receptor ionotropic NMDA 2B | XP_037869828.1 |
| Chordata | *Mus musculus* | Glutamate receptor ionotropic kainate 4 | AAI18011.1 |
|  | *Mus musculus* | Glutamate receptor 4 isoform 1 | NP 062665.4 |
|  | *Mus musculus* | Glutamate receptor ionotropic AMPA1 | EDL07794.1 |
|  | *Mus musculus* | Glutamate receptor ionotropic kainate 5 gamma 2 | AAI10683.1 |
|  | *Mus musculus* | Glutamate receptor ionotropic kainate 1 | Q60934.2 |
|  | *Mus musculus* | Glutamate receptor ionotropic kainate 3 | XP_006502835.1 |
|  | *Mus musculus* | Glutamate receptor ionotropic kainate 2 | NP_001104738.2 |
|  | *Mus musculus* | Glutamate receptor ionotropic NMDA 1 | NP_032195.1 |
|  | *Mus musculus* | Glutamate receptor ionotropic NMDA 3B | NP_569722.1 |
|  | *Mus musculus* | Glutamate receptor ionotropic NMDA 3A | NP_001263284.1 |
|  | *Mus musculus* | NMDA ionotropic glutamate receptor epsilon 4 | BAI44629.1 |
|  | *Mus musculus* | Glutamate receptor ionotropic NMDA 2C | NP_001418401.1 |
|  | *Mus musculus* | Glutamate receptor ionotropic NMDA 2B | Q01097.3 |
|  | *Mus musculus* | Glutamate receptor ionotropic NMDA 2A | NP_032196.2 |

**Table S4.** Primers used in this study

| Primer | Sequence (5’-3’) |
| --- | --- |
| SwPiezoF2 | GTGAAGTACGGTCTGGGTGG |
| SwPiezoR2 | TTTCTCGAAGCCGGCAATCT |
| SwTRPF1 | ATGGCGAATCCAAAGCCTCA |
| SwTRPR1 | TATCTCCGGCTCCGCTATCA |
| SwiGluRF1 | GTTCCCTCTCCCTGAGCAAC |
| SwiGluRR1 | TCGAAGTTTCCTGAGACGGC |
| SwActF1 | CCTCACCGAACGTGGTTACA |
| SwActR1 | GACCGTCGGGAAGTTCGTAG |
| SwTubF1 | TGTGTATCCCGCTCCTCAGA |
| SwTubR1 | GGAAGCCGTGATGGAACTCA |

**Table S5. Statistics of different cDNA libraries**

| Sample | Collection season | Raw reads | Base Number | Q30 |
| --- | --- | --- | --- | --- |
| A1 | Winter | 28585020 | 8,531,741,422 | 95.01% |
| A2 | Winter | 25,839,236 | 7,687,283,815 | 95.43% |
| A3 | Winter | 20,747,789 | 6,153,792,198 | 95.98% |
| S1 | Summer | 27,950,774 | 8,318,605,286 | 95.00% |
| S2 | Summer | 26,810,869 | 7,985,068,627 | 95.07% |
| S3 | Summer | 25,992,094 | 7,741,131,389 | 95.54% |

Table S6. Sensory protein genes in *S. wulingensis*

| Accession number | Gene | ORF (bp/aa) | Mw  (kDa) | pI | Number of TM | Best hit of Blastx | |
| --- | --- | --- | --- | --- | --- | --- | --- |
|  |  |  |  |  |  | \|Accession number\| protein name [species] e-value | Identity (%) |
| PQ202241 | SwPiezo1 | 7392/2463 | 283.95 | 8.30 | 38 | |XP_009020748.1| hypothetical protein HELRODRAFT_192356 [*Helobdella robusta*] 0.0 | 68.85% |
| PQ202242 | SwPiezo2 | Partail | / | / | / | |QZA74858.1| piezo [*Platynereis dumerilii*] 0.0 | 39.52% |
| PQ202243 | SwTRP1 | 4140/1379 | 157.53 | 6.95 | 6 | |XP_009010804.1| hypothetical protein HELRODRAFT_167034 [*Helobdella robusta*] 0.0 | 39.60 |
| PQ202244 | SwTRP2 | 3243 | 121.54 | 7.75 | 6 | |KAK2173436.1|hypothetical protein NP493_875g00020 [*Ridgeia piscesae*] 0.0 | 56.01 |
| PQ202245 | SwTRP3 | Partail | / | / | / | |XP_050400569.2| transient receptor potential cation channel subfamily A member 1 [*Patella vulgata*] 0.0 | 39.92 |
| PQ202246 | SwTRP4 | 4920 | 189.18 | 6.00 | 6 | |XP_009013475.1| hypothetical protein HELRODRAFT_190762 [*Helobdella robusta*] 0.0 | 36.64 |
| PQ202247 | SwTRP5 | 4833 | 184.63 | 6.81 | 6 | |XP_009013475.1| hypothetical protein HELRODRAFT_190762 [*Helobdella robusta*] 0.0 | 58.97 |
| PQ202248 | SwTRP6 | Partail | / | / | / | |XP_013397522.1| transient receptor potential cation channel subfamily M member 1-like isoform X2 [*Lingula anatina*] 0.0 | 45.04 |
| PQ202249 | SwTRP7 | Partail | / | / | / | |XP_013397522.1| putative transient receptor potential channel 1 [*Hirudo verbana*] 0.0 | 79.65 |
| PQ202250 | SwTRP8 | Partail | / | / | / | |AWJ68222.1| putative transient receptor potential channel 3 [*Hirudo verbana*] 0.0 | 91.20 |
| PQ202251 | SwiGluR1 | Partail | / | / | / | |AWJ68198.1| putative ionotropic glutamate receptor kainate-like 1 [*Hirudo verbana*] 0.0 | 85.71 |
| PQ202252 | SwiGluR2 | 2787 | 104.46 | 6.42 | 3 | |ARJ36889.1| glutamate receptor 2 [*Hirudo verbana*] 0.0 | 89.27 |
| PQ202253 | SwiGluR3 | Partail | / | / | / | |AWJ68208.1| putative ionotropic glutamate receptor kainate-like 9 [*Hirudo verbana*] 0.0 | 85.88 |
| PQ202254 | SwiGluR4 | 2880 | 108.23 | 6.22 | 3 | |AWJ68202.1| putative ionotropic glutamate receptor kainate-like 5 [*Hirudo verbana*] 0.0 | 81.16 |
| PQ202255 | SwiGluR5 | 3102 | 115.47 | 7.18 | 3 | |AWJ68200.1| putative ionotropic glutamate receptor kainate-like 3 [*Hirudo verbana*] 0.0 | 81.53 |
| PQ202256 | SwiGluR6 | 2985 | 113.10 | 7.59 | 3 | |AWJ68204.1| putative ionotropic glutamate receptor kainate-like 7 [*Hirudo verbana*] 0.0 | 86.48 |
